# Avoiding overflow metabolite formation in *Komagataella phaffii* fermentations to enhance recombinant protein production

**DOI:** 10.1186/s13036-024-00453-0

**Published:** 2024-10-03

**Authors:** Thomas Steimann, Judith Wegmann, Monica I. Espinosa, Lars Mathias Blank, Jochen Büchs, Marcel Mann, Jørgen Barsett Magnus

**Affiliations:** 1 AVT - Biochemical Engineering, RWTH Aachen University, Forckenbeckstraße 51, Aachen, 52074 Germany; 2 iAMB - Institute of Applied Microbiology, RWTH Aachen University, Worringer Weg 1, Aachen, 52074 Germany

**Keywords:** Crabtree effect, Overflow metabolite, Ethanol, *Pichia pastoris*, *Komagataella phaffii*, Stirred tank reactor, Recombinant protein production

## Abstract

**Background:**

*Komagataella phaffii* (*K. phaffii*), formerly known as *Pichia pastoris*, is a widely utilized yeast for recombinant protein production. However, due to the formation of overflow metabolites, carbon yields may be reduced and product recovery becomes challenging. This study investigates the impact of oxygen availability, different glucose concentrations and feeding strategies on overflow metabolite formation and recombinant protein production in *K. phaffii*.

**Results:**

High glucose concentrations in batch fermentation, as applied in literature, lead to substantial ethanol accumulation, adversely affecting biomass yield and product formation. Increasing dissolved oxygen setpoints does not significantly reduce ethanol formation, indicating that glucose surplus, rather than oxygen availability, drives overflow metabolism. Decreasing the initial glucose concentration to 5 g/L and adapting the feeding strategy of the fed-batch phase, effectively mitigates overflow metabolite formation, improving biomass yield by up to 9% and product concentration by 40% without increasing process time.

**Conclusions:**

These findings underscore the importance of a suitable glucose-feeding strategy in *K. phaffii* fermentation processes and highlight the detrimental effects of overflow metabolites on productivity. By optimizing carbon source utilization, it is possible to enhance fermentation efficiency and recombinant protein production with *K. phaffii*.

**Supplementary Information:**

The online version contains supplementary material available at 10.1186/s13036-024-00453-0.

## Background

*Komagataella phaffii* (*K. phaffii*), formerly known as *Pichia pastoris*, has emerged as a highly regarded organism for its efficient secretion of recombinant proteins, versatile post-translational modification capabilities, and ease of genetic manipulation, making it a preferred system for various industrial applications [1, 2]. Key to the success of *K. phaffii* fermentation processes is the careful selection of carbon sources. Historically, pure methanol served as the primary carbon source, but its toxicity, safety risks, and environmental impact pose significant challenges to process efficiency and product quality [3]. For biomass growth in batch mode, glycerol, a by-product of biodiesel production, has long been favoured due to its ability to support robust growth without inducing unwanted by-products or cellular aggregates [4]. However, glycerol usage presents its own challenges, including potential variations in purity and availability, which can impact process reproducibility and scalability.

In recent years, there has been a growing interest in employing glucose as the carbon source for *K. phaffii* fermentation. Glucose offers several advantages over glycerol, including easier handling in continuous cultivation, reduced microbial heat production and oxygen demand, as well as increased cell viability and lower protease release [5, 6]. Resulting lower cooling and oxygen requirements are great advantages from an operational point of view without sacrificing productivity losses [4]. Additionally, glucose-based expression systems have demonstrated lower contamination of heterologous proteins with host cell proteins, indicating potential improvements in product quality [5]. However, the choice between glycerol and glucose as carbon sources is not straightforward, as it depends on various factors such as process requirements, downstream processing considerations, and product characteristics. While many studies advocate for glucose as the superior substrate [7, 8], others report conflicting results, suggesting glycerol’s efficacy in supporting optimal fermentation conditions [9, 10].

A major disadvantage of glucose utilisation is ethanol accumulation during the initial batch phase under respirofermentative catabolism. Despite *K. phaffii* being classified as a Crabtree-negative yeast, ethanol formation occurs across a wide range of batch glucose concentrations ranging from 10 g/L to 50 g/L [11–20]. This leads to diminished biomass and product yields [4]. Furthermore, high ethanol concentrations and other overflow metabolites adversely affect the productivity of heterologous proteins in *K. phaffii* high-cell density fermentations. In methanol-induced mixed-carbon (glucose-methanol) cultivations with Mut^S^ strains, ethanol and acetate are preferentially metabolized before methanol [21]. While decreasing the flux towards biomass and product formation, ethanol and acetate act as repressors for the P_*AOX1*_ promoter, leading to a prolonged fermentation process due to a delayed induction [21–24]. It has been observed that ethanol levels as low as 50 mg/L can lead to significant repression of gene expression, emphasising the importance of preventing ethanol formation to maintain optimal induction conditions and maximise protein production [25, 26]. Moreover, ethanol toxicity at high concentrations is characterised by repression of key enzymes and metabolic pathways as well as effects on post-translational modifications [24]. Thus, preventing by-product formation is crucial for achieving high carbon yields and producing high-quality products by fully oxidizing the carbon source and maintaining a respiratory metabolism.

This study explores the phenomenon of overflow metabolite formation in the *K. phaffii* Mut^S^ BSYBG11 strain, investigating diverse strategies to mitigate their formation at a process level. It examines the interplay between oxygen availability, glucose surplus, and their impact on the production of a model recombinant protein. The methanol-induced P_*AOX1*_ promoter in a Mut^S^ strain was chosen as a model production system due to its high productivity and relevance in the literature [27].

## Materials and methods

### Strains

The following two strains were used for experiments: First, *K. phaffii* Mut^S^ BSYBG11 (BG11) obtained from Bisy GmbH (Hofstaetten a. d. Raab, Austria). This strain is a derivative of strain *K phaffii* NRRL Y-11,430/CBS 7435.

Second, strain BG11 was used as host for the genomic integration of an expression cassette coding for a recombinant structural protein under the control of the P_*AOX1*_ promoter. This strain is referred to as production strain in the manuscript. The production strain contains four target gene expression cassettes in chromosome 2 between genes 0456 and 0457. The nucleotide sequence of the native structural protein was codon optimized for *K. phaffii* to increase product titers.

### Media

All chemicals applied for media preparation were of analytical grade and purchased from Carl Roth GmbH (Karlsruhe, Germany) unless stated differently.

For shake flask precultures and Respiratory Activity Monitoring System (RAMOS) shake flask cultivations (flush phase 15 min, measuring phase 5 min), the *K. phaffii* strains were grown in mineral Syn6-MES medium [28]. The basic Syn6-MES medium consisted of 1.0 g/L KH_2_PO_4_, 7.66 g/L (NH_4_)_2_SO_4_, 3.3 g/L KCl, 3.0 g/L MgSO_4_ × 7H_2_O, 0.3 g/L NaCl, 39 g/L (0.2 M) 2-(N-morpholino)-ethanesulfonic acid (MES). All medium components were dissolved in deionized water, the pH was adjusted to 6.0 with 1 M NaOH and the medium was sterilized via autoclaving (121 °C for 20 min). Prior to use, the basic medium was supplemented with 10 mL/L of 100 g/L CaCl_2_ (sterile filtered), 10 mL/L of a 100 x micro-elements stock solution (sterile filtered), 10 mL/L of a 100 x vitamin stock solution (sterile filtered), 10 mL/L of a 100 x trace-elements solution (sterile filtered), and 20 mL/L (if not stated otherwise) glucose stock solution prepared with a concentration of 500 g/L (autoclaved). The stock solutions had the following compositions: micro-element stock solution: 6.65 g/L EDTA (ethylenediamine tetraacetic acid disodium sulfate), 6.65 g/L (NH₄)₂Fe(SO₄)₂×6H_2_O, 0.55 g/L CuSO_4_ × 5H_2_O, 2 g/L ZnSO_4_ × 7H_2_O and 2.65 g/L MnSO_4_×H_2_O. Vitamin stock solution: 0.04 g/L d-biotin and 13.35 g/L thiamine chloride. The d-biotin was dissolved in 10 mL of a (1:1) mixture of 2-propanol and deionized water. Thiamin chloride was dissolved separately in 90 mL deionized water. Afterwards, the two solutions were mixed. Trace element stock solution: 0.065 g/L NiSO_4_ × 6H_2_O, 0.065 g/L CoCl_2_ × 6H_2_O, 0.065 g/L H_3_BO_3_, 0.065 g/L KI, and 0.065 g/L Na_2_MoO_4_ × 2H_2_O.

For stirred tank bioreactor cultivations a medium proposed by Hyka et al. [13] was prepared. The basic medium consists of 7.23 g/L H_3_PO_4_, 0.64 g/L KOH, 0.17 g/L CaSO_4_ × 2H_2_O, 2.86 g/L K_2_SO_4_, 2.3 g/L MgSO_4_ × 7H_2_O and 0.1 mL/L polypropylene glycol (PPG). The basic medium solution was sterilized via autoclaving (121 °C for 20 min) and a 640 g/L glucose solution was added to a final concentration of 5–40 g/L. The medium was supplemented with 0.62 mL/L vitamin stock solution from Syn6-MES medium and 0.74 mL/L filter sterilized modified trace element solution PTM1. It consisted of 3.84 g/L CuSO_4_ × 5H_2_O, 0.08 g/L NaI, 3 g/L MnSO_4_×H_2_O, 0.2 g/L Na_2_MoO_4_ × 2H_2_O, 0.02 g/L H_3_BO_3_, 0.92 g/L CoCl_2_ × 6H_2_O, 20 g/L ZnCl_2_, 65 g/L FeSO_4_ × 7H_2_O and 5 mL/L 69% H_2_SO_4_. The medium components were dissolved in deionized water. The pH of the medium was titrated to 6.0 using an ammonia solution (30 vol%). Production was induced with 1 vol% methanol (purity > 99.5%).

The feed for bioreactor cultivations consisted of a 640 g/L glucose solution. The feed was supplemented with 12 mL/L PTM1 and 10 mL/L vitamin stock solution from Syn6-MES medium. After methanol induction of the bioreactor with 1 vol%, the feed was also supplemented with 7 vol% methanol to maintain a constant amount of ~ 1 vol% in the reactor. This reduces the glucose concentration in the feed to 570 g/L.

### Preculture

For bioreactor cultivations, a preculture was grown in four unbaffled 250 mL shake flasks with a filling volume of 10 mL. The flasks were inoculated with 100 µL glycerol stock cell suspension stored at -80 °C (optical density measured at 600 nm OD_600_ = 5) and cultivated for 18 h in a temperature-controlled hood (Climo-Shaker ISF1-X, Kuhner, Birsfelden, Switzerland) at 30 °C with a shaking frequency of 350 rpm and a shaking diameter of 50 mm.

### Main culture

Fermentations were performed in a 2 L Sartorius BIOSTAT^®^ stirred tank reactor (Sartorius, Göttingen, Germany) equipped with 4 baffles and two 6-bladed Rushton turbines (58 mm diameter and 11 mm height) mounted at heights of 30 mm and 90 mm from the bottom. A peristaltic pump (101 U/R, Watson-Marlow Pump Group, Falmouth, UK) was used for the feed. The supplementary data illustrates the experimental set-up (Supplementary Figure S1, Additional File 1). The fermentation process was adapted from the Invitrogen protocol [29]. The fed-batch phases, originally consisting of constant feeds, were replaced with open-loop exponential feeding profiles, as recommended in comparable studies [4, 15, 30] and detailed below. Cultivation was started in batch mode after inoculation to a starting OD_600_ of 0.2. Fermentation experiments were performed with an initial filling volume of 900 mL. If not stated otherwise, after glucose depletion (spike in the dissolved oxygen tension) at $\:{t}_{1}$, the first feed was started as a pre-programmed carbon limiting exponential feed with a pre-set growth rate µ_SET_ of 0.2 1/h to further increase biomass concentration at a growth rate near the maximal growth rate of the cells. A maximal growth rate on glucose in mineral media of 0.25 1/h is given in literature [31]. The feeding rate F_1_ is calculated by applying Eq. 1 for a set constant growth rate µ_SET_, given by Looser et al. [32]. Y_X/S_ is the biomass yield, m_S_ the maintenance coefficient, V_0_ the filling volume and X_0_ the biomass concentration at the start of the feed t_0_ and S_F_ the carbon concentration of the feed.

1 $$\:F\left(t\right)={\left(\frac{{\mu\:}_{SET}}{{Y}_{X/S\:}}+{m}_{S}\right)\cdot\:\frac{{V}_{0}\cdot\:{X}_{0}}{{S}_{F}}\cdot}\:e^{{\mu}_{SET}\:\left({t-t}_{0}\right)}$$

Based on previous experiments (data not shown), a biomass yield Y_X/S_ of 0.57 g/g and a maintenance coefficient m_S_ of 0.019 g/g/h is used for feed calculation. These values are in good accordance with literature [33–35]. The applied feed F_1_ for fermentations after a batch glucose concentration of 40 g/L is calculated from Eq. 1 and given in Eq. 2.

2 $$\:{F}_{1}=11\:mL/h\:{\cdot\:\:\:e}^{0.2\:{h}^{-1}\:\left({t-t}_{1}\right)}$$

After 5 h of the start of feed F_1_, at $\:{t}_{2}$, the cells were induced with 1 vol% methanol and the feeding rate reduced to F_2_ to reduce the growth rate µ_SET_ to 0.05 1/h. The feed rate is calculated with Eq. 1 and given in Eq. 3.

3 $$\:{F}_{2}=7\:mL/h\:{\cdot\:\:\:e}^{0.05\:{h}^{-1}\:\left({t-t}_{2}\right)}$$

If the initial batch was started with 5 g/L glucose, a lower biomass X_0_ at the start of the feed is expected. Therefore, the first feed was reduced accordingly. A feed rate of F_1+_ (calculated with Eq. 1) was applied for 15 h and is given in Eq. 4. The induction and second feed were kept unchanged. The supplementary data qualitatively illustrates the fermentation protocol (Supplementary Figure S2, Additional File 1).

4 $$\:{F}_{{1}^{+}}=1.2\:mL/h\:{\cdot\:\:\:e}^{0.2\:{h}^{-1}\:\left({t-t}_{1}\right)}$$

The observed growth rate µ was determined for all fermentations calculated from the absolute cell mass CDW_abs_. The growth rate was determined from a linear fit to the CDW_abs_ data in each section of the fermentation. The results are presented in the Supplementary Figure S4, Additional File 1.

The temperature was controlled at 28 °C, pH (EasyFerm Plus K8 225, Hamilton, Hoechst, Germany) at 6.0 with ammonia solution (30 vol%) and dissolved oxygen tension (VisiFermTM DO 225 pO2 sensor, Hamilton, Hoechst, Germany) at 30% air saturation by cascade control of stirring rate (500–1500 rpm) and aeration rate (1–3 sL/min).

### Offline analysis

Samples were taken for offline analysis on a regular basis and measured in triplicates. The OD_600_, cell dry weight (CDW) and target protein concentration were determined. OD_600_ was measured using a Genesys 20 photometer (Thermo Scientific, Darmstadt, Germany). Samples were diluted with 0.9% (w/v) NaCl, if necessary. CDW was determined gravimetrically in triplicates in 2 mL reaction tubes. Samples were centrifuged at 18,000 rcf for 10 min and the supernatant was filtered with a 0.2 μm cut-off filter (Millipore-Sigma, Burlington, USA). Protein concentration was determined using size exclusion chromatography (GPC EcoSEC, Tosoh Bioscience GmbH, Stuttgart, Germany) equipped with 3 PROTEEMA columns (PSS Polymer, Mainz, Germany) and a UV detector (214 nm). The mobile phase consisted of 0.2 M phosphate buffer at a pH of 5.3 with a flow rate of 1 mL/min. The temperature was set to 40 °C. For calibration 2 g/L BSA (bovine serum albumin) was used. Glucose, methanol and ethanol concentrations were determined using high-performance liquid chromatography (HPLC Dionex UltiMate 3000, Thermo Scientific, Darmstadt, Germany) equipped with an organic acid column at 80 °C. The mobile phase consisted of 50 mM H_2_SO_4_ with a flow rate of 0.8 mL/min.

### Calculations

Yields on substrate were calculated for all fermentations. Biomass yield Y_X/S_ was determined according to Eq. 5. The total biomass X_abs_ was determined from the biomass at the end of the fermentation X_end_ and the final filling volume V_end_. The total fed glucose mass was determined from the initial glucose concentration S_0_, the feed concentration of the first feed S_F,1_ and the second feed S_F,2_. V_0_ is the initial filling volume, V_F,1_ is the volume of the first feed and V_F,2_ is the volume of the second feed. The fed volumes were tracked gravimetrically. The feed density was determined as 1.23 kg/L.

5 $$\:{Y}_{X/S}=\frac{{X}_{abs}}{{S}_{abs}}=\frac{{X}_{end}\cdot\:{V}_{end}}{{S}_{0}\cdot\:{V}_{0}+{S}_{F,1}\cdot\:{V}_{F,1}+{S}_{F,2}\cdot\:{V}_{F,2}}\:\:[g/g]$$

Product yield Y_P/S_ was analogously determined according to Eq. 6. The total product mass P_abs_ was corrected by the volume fraction of the supernatant v_supernatant_ determined as 0.6 L/L.

6 $$\:{Y}_{P/S}=\frac{{P}_{abs}}{{S}_{abs}}=\frac{{P}_{end}\cdot\:{V}_{end}\cdot\:{v}_{supernatant}}{{S}_{0}\cdot\:{V}_{0}+{S}_{F,1}\cdot\:{V}_{F,1}+{S}_{F,2}\cdot\:{V}_{F,2}}\:[g/g]$$

The respiratory quotient (RQ) is calculated from the oxygen and carbon dioxide transfer rates (OTR and CTR) according to Eq. 7.

7 $$\:RQ=\frac{CTR}{OTR}\:[-]$$

The oxygen consumption can be calculated from the OTR integral according to Eq. 8.

8 $$\:{n}_{{O}_{2}}=\underset{{t}_{0}}{\overset{t}{\int\:}}OTR\left({t}^{{\prime\:}}\right)\:dt{\prime\:}\:[mol/L]$$

For the elemental carbon balance, the total produced CO_2_ was determined from the integral of the carbon dioxide transfer rate (CTR) measured with an off-gas analyser (DASGIP GA4, Eppendorf, Hamburg, Germany) according to Eq. 9. The dynamic filling volume V(t) due to the feed was considered for the integration. M(CO_2_) denotes the molar mass of carbon dioxide.

9 $$\:{m}_{{CO}_{2}}=\:{M}_{{CO}_{2}}\cdot\:\underset{{t}_{0}}{\overset{{t}_{end}}{\int\:}}CTR\left({t}^{{\prime\:}}\right)\cdot\:V\left({t}^{{\prime\:}}\right)\:dt{\prime\:}\:\left[g\right]$$

For growth without production or overflow metabolite formation, the following elemental balance given in Eq. 10 was considered. The elemental balance for the biomass for the BG11 strain was determined elsewhere [36].

10 $$\:{C}_{6}{H}_{12}{O}_{6}+0.58\:N{H}_{3}+2.2\:{O}_{2}\to\:3.6\:{C}_{1}{H}_{1.83}{O}_{0.56}{N}_{0.16}+2.4\:C{O}_{2}+3.57\:{H}_{2}O$$

The resulting respiratory quotient (RQ) can be calculated as 1.09 and the substrate-specific oxygen demand as 12.2 mmol_O2_/g_Glc_.

To determine the significance of the results a one-way ANOVA with a significance level of α = 0.05 was used (OriginPro 2022, OriginLab Corporation, Northampton, USA). Normal distribution was assumed and homogeneity of variance was determined using Levene´s test. Results are shown in Supplementary Figure S5, Additional File 1.

## Results and discussion

### High glucose batch concentration

**Fig. 1:**
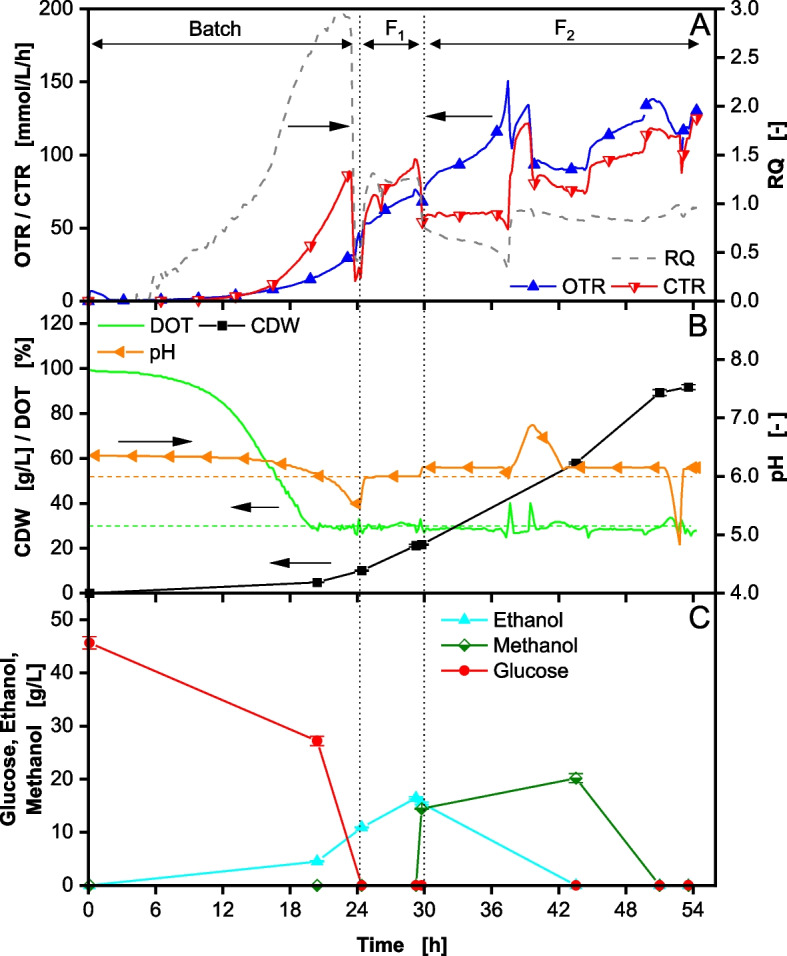
Fed-Batch fermentation of *Komagataella phaffii* BG11 strain with 40 g/L glucose in initial batch phase and DOT controlled > 30% performed in a 2 L stirred tank reactor. After 30 h (second vertical dotted line) induction is performed by methanol addition. In (**A**) the oxygen transfer rate (OTR), carbon dioxide transfer rate (CTR) and respiratory quotient (RQ) are depicted over time. In (**B**) the dissolved oxygen tension (DOT), cell dry weight (CDW) and pH are depicted over time. In (**C**) the glucose, ethanol and methanol concentrations are depicted over time. Cultivation was performed with 40 g/L initial glucose concentration. The start of feeding is marked by the first vertical dotted line. The feeding solution consisted of 640 g/L glucose. Induction by adding 1 vol% MeOH. 7 vol% MeOH was added to the feeding solution F_2_ after induction. Feeding rate F_1_ = 11 mL/h *exp(0.2 h^−1^ * t) from 25–30 h. Feeding rate F_2_ = 7 mL/h *exp(0.05 h^−1^ * t) from 30–54 h. The pH was controlled at 6

A reference fed-batch fermentation with 40 g/L glucose (46 g/L measured) at the start of the fermentation was conducted. The results are shown in Fig. 1.

During the initial batch phase, the oxygen transfer rate (OTR) and carbon dioxide transfer rate (CTR) increase exponentially as glucose is consumed (Fig. 1A). The dissolved oxygen tension (DOT) decreases and is controlled at 30% by a cascade control with the stirring speed and aeration rate (Fig. 1B). In this phase, a respiratory quotient (RQ) well above the calculated RQ of 1.09 (Eq. 10), is observed (Fig. 1A), indicating a strong overflow metabolite formation. A high concentration of ethanol could be measured (Fig. 1C). Other studies have shown the production of further metabolites from the respiro-fermentative pathway including pyruvate, acetaldehyde, acetate, and arabitol [11, 16, 21]. In the batch phase, the cell dry weight (CDW) reaches 10 g/L (Fig. 1B). A total of 11 g/L ethanol accumulates in the supernatant (Fig. 1C), leading to a biomass yield (Y_X/S_) of only 0.22 g/g. This yield is lower than usual for *K. phaffii* processes, using glucose as a carbon source [33–35]. However, when applying high glucose concentrations in batch mode, similar results are reported due to overflow metabolite formation [4, 15]. After the initial batch phase, the first exponential feed F_1_ is started, to further increase biomass concentration prior to induction and derepress the P_*AOX1*_ promoter [37]. An exponential growth rate of 0.2 1/h, close to the maximal growth rate for *K. phaffii* [31], is set through a preprogrammed feed (Eq. 2). During this phase, the OTR and CTR continue to rise. The RQ drops to 1.3, still indicating some overflow metabolite formation (RQ > 1.09 from Eq. 10). This is confirmed by the rise in ethanol concentration to 16.5 g/L (Fig. 1C). A CDW of 22 g/L is achieved before feed F_2_ is initiated. This CDW is lower than anticipated, likely due to the elevated ethanol concentration. This hypothesis is supported by the observed growth rate µ of 0.17 1/h (Supplementary Figure S4A, Additional File 1), which is lower than the set growth rate of 0.2 1/h by the feed. Consequently, a portion of the glucose is diverted from biomass production to the formation of overflow metabolite. Although the applied BG11 host strain does not express a recombinant protein, methanol induction was performed through a methanol pulse of 1 vol% (14 g/L measured) after 30 h. To compensate for methanol consumption and evaporation and to keep the methanol concentration in the reactor constant, the feed was also complemented with 7 vol% methanol. The feed rate was reduced and followed the second pre-programmed exponential feed F_2_ with a growth rate of 0.05 1/h (Eq. 3). At the start of the induction phase, the CTR drops significantly, due to the reduced glucose availability. However, the OTR continues to rise, leading to an RQ drop from 0.7 to 0.3 from 30 h until 37.5 h. This suggests the metabolization of the previously formed overflow metabolites. At 37.5 h, the RQ increases to ~ 1.0 and the pH rises to 6.9 (Fig. 1B), indicating the metabolization of an acidic overflow metabolite, possibly acetate, as it matches the measured RQ (refer to supplement material for theoretical RQ calculation, Additional File 1). Acetate was already determined as an overflow metabolite for *K. phaffii* [38, 39]. Additionally, ethanol was not detectable after 43.5 h. Similar patterns of ethanol production and re-assimilation have been reported in previous studies [21, 22, 40–43]. Vanz et al. [44] as well as Karaoğlan et al. [14] could also identify the occurrence of two alcohol dehydrogenases, that are both responsible for the production and consumption of ethanol and may have additional functions that are not yet fully understood [14, 44]. The results reemphasize that overflow metabolites accumulate during the batch and high-growth fed-batch phases when glucose is provided as the carbon source. High uptake rates lead to a redirected flux towards the fermentative pathway to avoid NADH accumulation when the respiration system becomes saturated, as assumed for the methylotrophic yeast *Hansenula polymorpha* [45].

After 39.5 h, the pH starts to drop again and continues to be regulated at pH 6 by ammonia addition. The RQ drops to 0.8–0.9, indicating the simultaneous consumption of glucose and methanol (refer to supplement material for theoretical RQ calculation, Additional File 1). To this point, likely no methanol is consumed, as can be seen by the accumulation of the methanol additionally introduced via the feed. Ethanol has been shown to inhibit methanol consumption [21]. Further, the methanol concentration decreases only after ethanol is consumed, reaching the detection limit after 51 h. At this time, the RQ rises to ~ 1, reaffirming the methanol metabolization [46]. Therefore, the fed methanol was not sufficient to keep a constant concentration of 1 vol% in the reactor. On-line methanol control strategies could be applied to prevent this issue [47, 48]. The fermentation is stopped after 54 h, reaching a total CDW of 92 g/L from a cumulated glucose concentration of 165 g/L. This results in a total biomass yield relative to glucose of 0.56 g/g, comparable with previous results [33–35].

Thermodynamically, it is not reasonable to allow for overflow metabolite formation and consumption, as exergy is being lost in the process. Therefore, the formation of up to 16 g/L ethanol should be avoided. Furthermore, while the introduction of the first feeding phase reduces promoter repression by glucose prior to induction, the accumulation of overflow metabolites, such as ethanol, strongly represses product formation [21]. In the shown reference fermentation, ethanol is present in the first 9.5 h of the production phase, likely leading to a potential productivity loss, since energy is redirected from product formation.

### Increase of dissolved oxygen setpoint

**Fig. 2:**
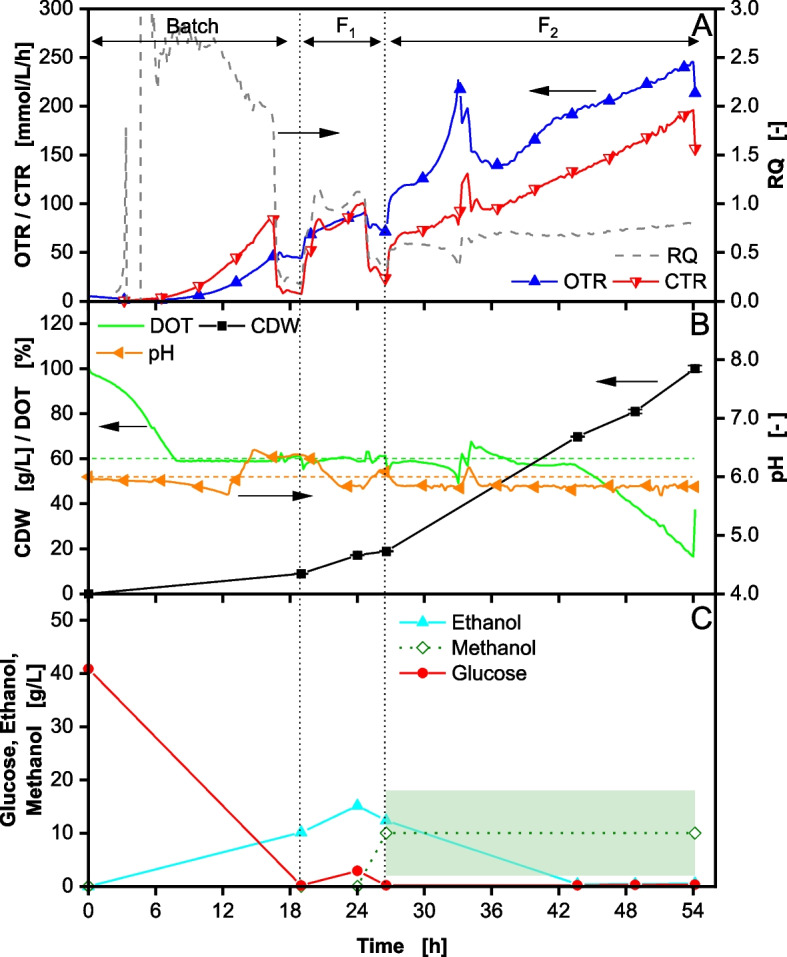
Fed-Batch fermentation of *Komagataella phaffii* BG11 strain with 40 g/L glucose in initial batch phase and DOT controlled > 60% performed in a 2 L stirred tank reactor. After 27 h (second vertical dotted line) induction is performed by methanol addition. In (**A**) the oxygen transfer rate (OTR), carbon dioxide transfer rate (CTR) and respiratory quotient (RQ) are depicted over time. In (**B**) the dissolved oxygen tension (DOT), cell dry weight (CDW) and pH are depicted over time. In (**C**) the glucose and ethanol concentrations are depicted over time. Methanol concentration is given as theoretical values (background shadow). Cultivation was performed with 40 g/L initial glucose concentration. The start of feeding is marked by the first vertical dotted line. The feeding solution consisted of 640 g/L glucose. Induction by adding 1 vol% MeOH. 7 vol% MeOH was added to the feeding solution F_2_ after induction. Feeding rate F_1_ = 11 mL/h *exp(0.2 h^−1^ * t) from 19–24 h. Feeding rate F_2_ = 7 mL/h *exp(0.05 h^−1^ * t) from 27–54 h. The pH was controlled at 6

To address ethanol formation, oxygen availability was investigated. Although hypoxia has been reported beneficial for the expression of foreign genes in *K. phaffii* [47], especially for Mut^+^ phenotypes, it is associated with increased overflow metabolism [49, 50]. Conversely, excess oxygen has also been shown to promote the formation of specific proteins in *K. phaffii* Mut^S^ phenotypes [51, 52]. To assess the influence on overflow metabolite formation, the DOT setpoint was increased from 30% to 60% during a fermentation. The initial glucose concentration was kept at 40 g/L (41 g/L measured). The results are shown in Fig. 2.

The increase in oxygen availability did not significantly reduce ethanol formation in the batch phase. The slightly lower glucose concentration of 41 g/L in the batch (compared to 46 g/L in Fig. 1) led to a CDW of 9 g/L (Fig. 2B) and an ethanol concentration of 10 g/L after glucose consumption at 19 h (Fig. 2C). The biomass yield Y_X/S_ of 0.22 g/g is similar to the reference. During the first feed phase, the RQ is lower than for the reference fermentation (Fig. 2A), indicating fewer overflow metabolites being produced. Although ethanol formation is comparable, reaching a concentration of 15 g/L at 24 h (Fig. 2B), the formation of acidic overflow metabolites is reduced. The pH peak corresponding to the consumption of these acids (Fig. 2B) only reaches a pH of 6.2 (instead of 6.9 in Fig. 1B), suggesting that higher oxygen availability shifts by-product formation from oxidized metabolites like acids to more reduced metabolites like ethanol and arabitol. Moreover, the observed growth rate during the growth phase does not reach the growth rate set by the feed of 0.2 1/h (Supplementary Figure S4B, Additional File 1), indicating that a portion of the fed glucose is diverted away from biomass formation. As per the reference, the cells were induced by a methanol pulse and supplementation of F_2_ with methanol. Similar ethanol consumption patterns can be observed to those in the reference cultivation (Fig. 2C). Due to malfunction of the HPLC, no precise quantification of methanol could be performed. However, the RQ value during the induction phase remains at ~ 0.8, indicating the continuous consumption of methanol until the end of the fermentation, suggesting a non-limiting methanol concentration during the induction phase. After 43 h, the maximal oxygen mass transfer coefficient (k_L_a) of the reactor is reached resulting in a subsequent DOT decrease. The fermentation was stopped when the DOT reached 15%.

In summary, the increase of the DOT setpoint to 60% did not result in a significant reduction in ethanol formation. Therefore, ethanol formation in *K. phaffii* is not linked to limiting oxygen availability, but probably to surplus glucose uptake, interpreted as a consequence of the crabtree effect similar to *S. cerevisiae* and *E. coli* [53–55].

### Decrease of glucose batch concentration

**Fig. 3:**
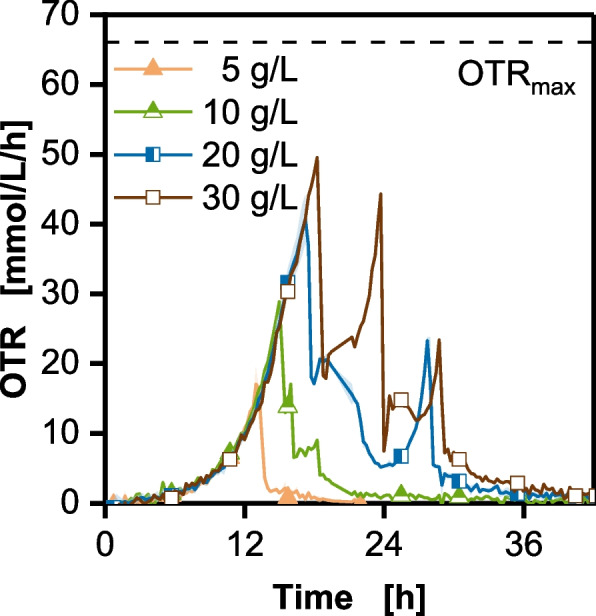
Variation of initial glucose concentration of batch cultivations of *Komagataella phaffii* BG11 strain in shake flasks. Cultivation was performed in a temperature-controlled shaker at 30 °C, shaken at 350 rpm with a shaking diameter d_0_ of 50 mm. OTR was monitored with an in-house build RAMOS device every 20 min (flush phase 15 min, measuring phase 5 min). Only every 20th data point is shown as a symbol for simplicity. The lines are drawn through all measured data points. An OTR_max_ of 66 mmol/L/h was calculated with the correlation by Meier et al. (2016) and is shown as a dashed line

To test the hypothesis that overflow metabolite formation is linked to glucose surplus and determine the maximal glucose concentration tolerable to prevent ethanol formation, a batch cultivation varying the initial glucose concentration was performed in shake flasks. The initial glucose concentration was varied from 5 g/L to 30 g/L. The cultivation was monitored using the in-house developed RAMOS device [56, 57], commercially available as Transfer rate Online Measurement (TOM) device (Adolf Kühner AG, Birsfelden, Switzerland). Both systems monitor the OTR online during the cultivation. The results can be seen in Fig. 3.

All cultures grow exponentially until glucose is depleted, marked by the drop after the first OTR peak [56]. For 10 g/L, 20 g/L and 30 g/L glucose, further peaks in the OTR can be seen, indicating the successive consumption of different overflow metabolites, produced during previous glucose uptake. Since ethanol, acetate and arabitol were probably produced in the previous stirred bioreactor cultivations (Figs. 1 and 2), it is most likely that these metabolites were formed here too. Ethanol may be associated with the second OTR peak, consistent with previous studies, demonstrating ethanol consumption prior to acetate in *K. phaffii* [21]. Furthermore, the OTR integral of the glucose peak is not proportional to the amount of glucose employed and does not match the calculated stoichiometry (refer to Supplementary Figure S3, Additional File 1), reaffirming the overflow metabolite formation during glucose consumption. To exclude the impact of oxygen availability on the overflow metabolite formation, the maximum oxygen transfer capacity OTR_max_ of the shake flask system was calculated with the correlation by Meier et al. [58] and is shown in Fig. 3 as a dashed line. None of the cultures reach the calculated OTR_max_ of 66 mmol/L/h. The absence of an OTR plateau further supports the conclusion that an oxygen limitation is not occurring [56]. In other words, all cultures have sufficient oxygen supply, confirming the results of Fig. 2, that increased oxygen availability does not lead to the elimination of overflow metabolite formation. For 10 g/L glucose in the batch, overflow metabolite formation is strongly reduced. Only a small overflow peak can be seen at 18 h. Lowered glucose concentrations result in lower glucose uptake rates, which are known to strongly influence the crabtree effect and its underlying mechanisms [19, 54]. Reducing the glucose concentration to 5 g/L completely eliminates overflow metabolite formation as indicated by missing additional OTR peaks. Additionally, the total oxygen consumption, calculated from the OTR integral (Eq. 8), of 50 mmol/L fits the theoretical stoichiometric prediction derived from Eq. 10 (refer to Supplementary Figure S3, Additional File 1), further confirming the results. A similar observation was made by Weis et al. [20] and Wollborn et al. [26]. The increase in glucose concentration from 2 g/L to 30 g/L did not lead to the expected stoichiometric increase in biomass.

**Fig. 4:**
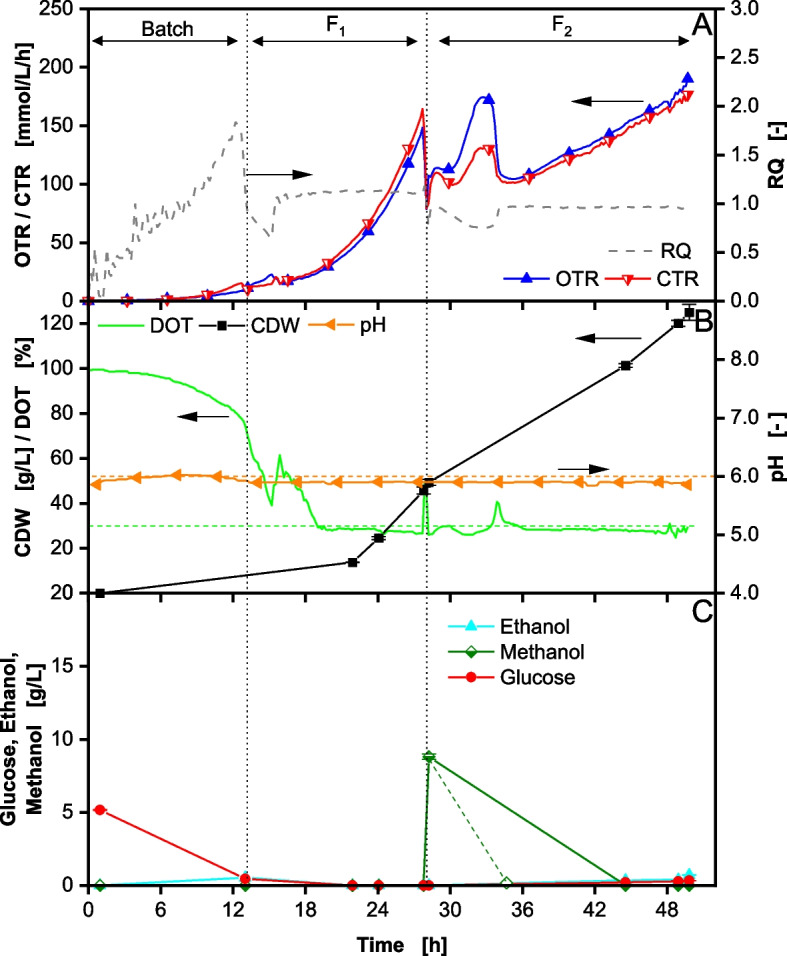
Fed-Batch fermentation of *Komagataella phaffii* BG11 strain with 5 g/L glucose in initial batch phase and DOT controlled > 30% performed in a 2 L stirred tank reactor. After 28 h (second vertical dotted line) induction is performed by methanol addition. In (**A**) the oxygen transfer rate (OTR), carbon dioxide transfer rate (CTR) and respiratory quotient (RQ) are depicted over time. In (**B**) the dissolved oxygen tension (DOT), cell dry weight (CDW) and pH are depicted over time. In (**C**) the glucose, ethanol and methanol concentrations are depicted over time. Cultivation was performed with 5 g/L initial glucose concentration. The start of feeding is marked by the first vertical dotted line. The feeding solution consisted of 640 g/L glucose. Induction by adding 1 vol% MeOH. 7 vol% MeOH was added to the feeding solution F_2_ after induction. Feeding rate F_1_ = 1.2 mL/h *exp(0.2 h^−1^ * t) from 13–28 h. Feeding rate F_2_ = 7 mL/h *exp(0.05 h^−1^ * t) from 27–50 h. The pH was controlled at 6

To reduce overflow metabolite formation in the stirred tank fermentation, the process is adapted by reducing the initial glucose concentration in the batch phase to 5 g/L, as deduced from Fig. 3. Furthermore, the first feed phase was modified to F_1+_ (Eq. 4) and prolonged to reach a comparable total glucose amount provided (74 g compared to 68 g in Fig. 1) and accumulated biomass before induction as in the reference process. The results can be seen in Fig. 4.

During the first 13 h, the initial glucose is consumed in batch mode and 0.45 g/L ethanol is formed (Fig. 4C). The RQ reaches a value of 1.8 (Fig. 4A), matching the formation of ethanol. Contrary to the shake flask experiment, some ethanol is still formed in the batch with 5 g/L glucose. This could be explained by the elevated osmolarity of the high cell density medium used for the stirred tank fermentations. Higher osmotic pressure was shown to lead to overflow metabolite formation [59, 60]. After glucose consumption and the start of the first feed F_1+_, the RQ drops below 1 (Fig. 4A), suggesting metabolization of the previously formed ethanol. After 2 h from the start of F_1+_ (29 h total process time), the RQ increases to 1.1 indicating growth on glucose (Eq. 10) and the complete consumption of ethanol [45]. Offline samples taken after 22 h confirm the absence of overflow metabolites in the supernatant (Fig. 4C). At 28 h, the cells are induced by spiking 1 vol% methanol (8.8 g/L measured) into the fermenter. Furthermore, the feed is reduced to F_2_, equivalent to Figs. 1 and 2. At this point, the RQ drops significantly to 0.8 (Fig. 4A), indicating the immediate start of methanol metabolization [46]. A spike in OTR and CTR can also be seen. Due to the absence of ethanol or other overflow metabolites in the hours before induction, the P_*AOX1*_ promoter is derepressed [37] and methanol consumption is not inhibited, as opposed to what is shown in Fig. 1. Further, no spike in the pH is seen as in Figs. 1 and 2. Therefore, after 34 h, when the RQ rises to 0.96, methanol is most likely fully consumed and becomes limiting (shown in Fig. 4C with a dashed line). Interestingly, during the last hours of the fermentation, small amounts of ethanol accumulated in the reactor, reaching a concentration of 0.7 g/L. This could be a consequence of the high cell density of 125 g/L achieved at the end of the fermentation, leading to oxygen inhomogeneities in the reactor due to mass transfer limitations [15, 61]. Nonetheless, by reducing the glucose concentration in the initial batch phase to 5 g/L, ethanol formation was significantly reduced and the biomass yield relative to glucose Y_X/S_ could be improved by 9.3% to 0.61 g/g (significant difference with *p* < 0.01). The results are consistent with previous findings by Hang et al. [19]. They also reported that lower glucose concentrations were beneficial for *K. phaffii*, as less ethanol was formed, while simultaneously increasing productivity.

### Application in a recombinant protein production process

**Fig. 5:**
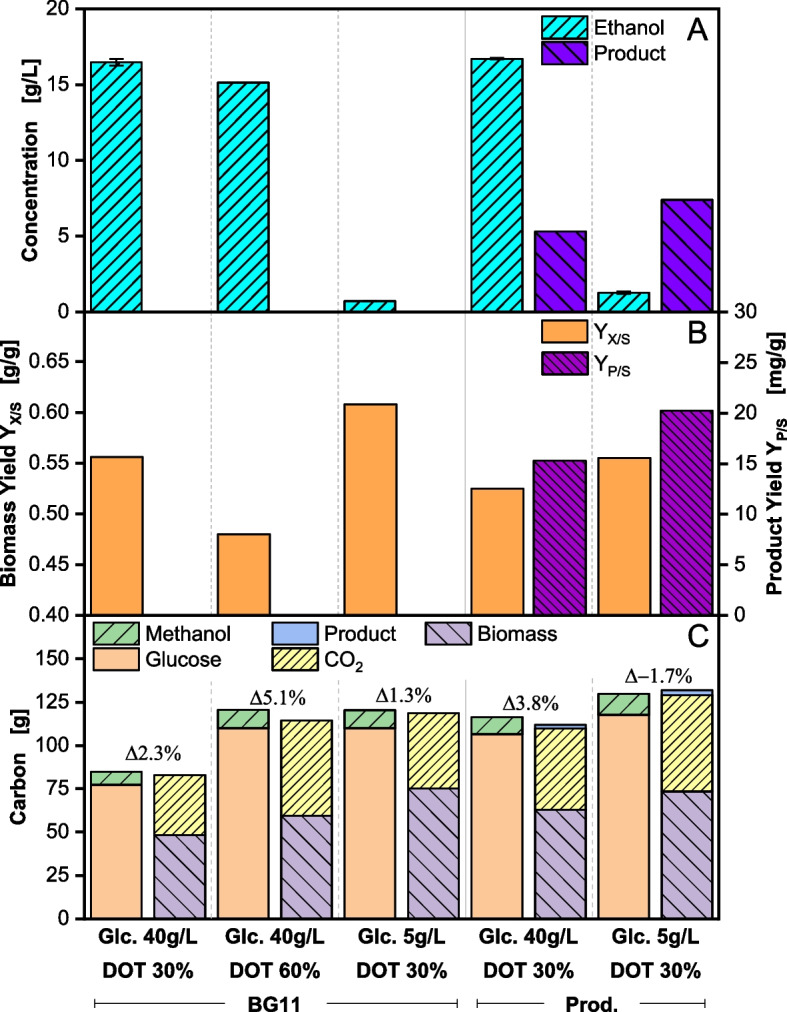
Comparison of *Komagataella phaffii* BG11 strain (BG11) and recombinant protein production strain (Prod.) in five different fermentations. For the BG11 strain the initial glucose concentration was set at 40 g/L (DOT > 30% and DOT > 60%) or 5 g/L (DOT > 30%). For the production strain, the initial concentration was set at 40 g/L or 5 g/L. The DOT was kept > 30%. (**A**) shows maximal ethanol and product formation, (**B**) substrate and product yield, (**C**) elemental carbon balance. The deviation between carbon influx (methanol, glucose) and carbon outflux (biomass, CO2, product) in (**C**) is given above the columns

Overflow metabolite formation can lead to productivity losses during recombinant protein production. On one side, exergy is lost during the formation and reassimilation of the metabolites. On the other side, overflow metabolites such as ethanol are known to repress methanol-induced promoters such as the P_*AOX1*_ promoter [21]. Therefore, two fermentations with a recombinant protein-producing strain were performed to quantify productivity losses due to overflow metabolite formation. The results are shown in Fig. 5.

Maximum ethanol and product concentration during the fermentations are compared in Fig. 5A. The first three bars (BG11) show the results from Figs. 1, 2 and 4 and are compared to a production strain (Prod.). Applying the fermentation protocol with an initial glucose concentration of 40 g/L for the production strain leads to a maximal ethanol concentration of 16.7 g/L (Glc. 40 g/L Prod. in Fig. 5A), comparable to 16.5 g/L for the BG11 strain (Glc. 40 g/L BG11. in Fig. 5A). Similar to the BG11 strain, an upward pH spike is seen (data not shown), suggesting also the formation of an undetected acid, assumed to be acetate (see Figs. 1 and 2). A total product concentration of 5.3 g/L (Glc. 40 g/L Prod. in Fig. 5A) could be achieved during the fermentation. This value is comparable to other secreted recombinant protein production processes with *K. phaffii* [62]. The reduction of the batch glucose concentration from 40 g/L to 5 g/L for the production strain also drastically reduced overflow metabolite formation (Glc. 5 g/L Prod. in Fig. 5A). A maximal ethanol concentration of 1.3 g/L is measured in this second fermentation with 5 g/L initial glucose, reducing overflow metabolite formation by 90%. More importantly, the product concentration increases by 40% from 5.3 g/L to 7.4 g/L (Glc. 5 g/L Prod. in Fig. 5A) confirming the assumption that the formation and reassimilation of overflow metabolites can reduce product titer. The adverse impact of overflow metabolites not only affects cellular growth but also the activity of the applied promoter P_*AOX1*_ [21]. As ethanol and acetate are known to repress the promoter, product formation remains inhibited even after induction with methanol (Glc. 40 g/L Prod. in Fig. 5A). The inhibitory effects of ethanol and acetate are particularly detrimental because they interfere with the transcriptional machinery required for high-level expression of the desired protein, ultimately impairing overall process performance. Additionally, the accumulation of these metabolites can disrupt the cellular redox balance, thereby inhibiting key biosynthesis pathways involved in protein synthesis, folding, and secretion [63]. Moreover, the metabolic stress induced by overflow metabolites triggers cellular responses diverting energy and resources away from protein synthesis and towards homeostasis and stress response mechanisms [64]. Similar inhibitory effects by ethanol have been observed for heterologous protein expression in *S. cerevisiae* and *E. coli* [65, 66]. In these organisms, the accumulation of ethanol can lead to stress responses that negatively impact cellular physiology, including reduced protein synthesis rates, altered membrane permeability, and increased oxidative stress. These stress responses can further diminish the efficiency of recombinant protein production, thereby reducing overall yield and quality.

Figure 5B summarizes biomass yield Y_X/S_ and product yield Y_P/S_ relative to glucose and Fig. 5C shows the elemental carbon balance for all fermentations. The carbon balance is closed. Small deviations < 5% could be explained by methanol evaporation not quantified in the fermenter off-gas. Interestingly, the increase of the DOT setpoint to 60% for the BG11 strain (Fig. 2) does not improve the biomass yield (significant difference with *p* < 0.001) but leads to higher maintenance and CO_2_ formation (Fig. 5C). As described before (Fig. 4), the reduction of the glucose concentration in the batch phase (Glc. 5 g/L BG11 in Fig. 5B) leads to an increase in biomass yield. The same is true for the production strain (Glc. 5 g/L Prod. in Fig. 5B). Here, an increase in yield by 5.6% from 0.53 g/g to 0.56 g/g can be seen (significant difference with *p* < 0.05). Further, the biomass yield of the production strain is 6–9% lower than for the empty vector strain BG11 (significant difference with *p* < 0.05). This is likely due to the presence of the gene expression cassette, which imposes a metabolic burden on the cells [67]. The increased carbon flux into maintenance for the production strain (Prod.) compared to the empty vector (BG11) is confirmed by the higher CO_2_ formation shown in the carbon balance (Fig. 5C). The expression of heterologous DNA can lead to cell stress and changes in metabolism, including a redistribution of carbon fluxes [68, 69]. The synthesis of heterologous proteins thereby lowers growth-related processes, linked to lower biomass yields [16, 70]. Regarding product yield Y_P/S_ (Fig. 5B), the reduced glucose concentration improves the yield by 31%, indicating that the change in carbon flux distribution had a direct impact on product formation.

## Conclusions

The methylotrophic yeast *Komagataella phaffii* has become one of the most important hosts for heterologous protein production. The exploitation of the P_*AOX1*_ promoter and methanol as inducer leads to a high translational capacity. However, the formation of overflow metabolites during the process inhibits efficient protein production. This challenge is addressed by optimizing the cultivation strategy to minimize overflow metabolism and enhance product formation.

The results showed that both strains, the Mut^S^ strain (BG11) lacking an expression cassette and the example production strain, exhibited overflow metabolism, as indicated by characteristic patterns in the RQ and the detection of ethanol concentrations of up to 16 g/L. The first attempt to reduce the formation of overflow metabolites was the increase of oxygen availability by raising the DOT setpoint. Despite increasing the DOT setpoint to 60%, ethanol formation remained largely unaffected, indicating that oxygen availability did not directly contribute to overflow metabolite formation, but was rather triggered by glucose surplus.

The influence of glucose concentration in the batch was tested in RAMOS shake flask cultivations and transferred to a stirred tank fed-batch process. By reducing the glucose concentration to 5 g/L in the initial medium, overflow metabolite formation was eliminated. This adjustment not only improved the biomass yield by 5.6–9.3% but also led to a notable increase in heterologous protein concentration by 40%, thereby enhancing the product yield by 25%. These findings underscore the importance of optimizing the glucose concentration and feeding strategy to mitigate overflow metabolite formation and maximize product yields in *K. phaffii* fermentation processes by increasing carbon fluxes toward biomass and product formation.

In conclusion, the study elucidates the detrimental effects of undesired overflow metabolite formation, particularly ethanol, during *K. phaffii* fermentation. Further, it proposes an optimized fermentation protocol to avoid overflow formation and improve productivity through practical and robust process engineering strategies.

## Abbreviations

**Table:** 

**Abbreviation**	**Explanation**		**Units**
CTR	Carbon dioxide transfer rate		mmol/L/h
F	Feed rate		ml/h
m_S_	Maintenance coefficient		g/g/h
OD_600_	Optical density at 600 nm		-
OTR	Oxygen transfer rate		mmol/L/h
P	Product concentration		g/L
RQ	Respiratory quotient		-
S_F_	Feed glucose concentration		g/L
t	Time		h
V	Volume		L
v_supernatant_	Volume fraction of supernatant		L/L
X	Biomass concentration		g/L
Y_X/S_	Biomass yield relative to glucose		g/g
Y_P/S_	Product yield relative to glucose		g/g
µ_SET_	Set constant exponential growth rate		1/h
**Indices**	**Explanation**		
abs	Absolute value		
0	Initial value at the start of the fermentation		
1	Value for feed 1		
2	Value for feed 2		
end	End value at the end of the fermentation		

## Supplementary Information

Additional file 1: Figure S1. Diagram of the experimental set-up, Figure S2. Fermentation protocol, Figure S3. Total oxygen consumption during glucose metabolization, Figure S4. Determination of growth rate µ, Figure S5. Statistical significance of pairwise comparison of biomass yields Y_X/S_, Calculation of theoretical RQ values.

## Data Availability

The datasets used and/or analyzed during the current study are available from the corresponding author on reasonable request.

## Data availability

No datasets were generated or analysed during the current study.
