# Supplementary material for: Avoiding overflow metabolite formation in Komagataella phaffii fermentations to enhance recombinant protein production
Source: J Biol Eng. 2024 Oct 3;18:54. doi: 10.1186/s13036-024-00453-0 (PMC11448000; doi:10.1186/s13036-024-00453-0)
Supplement: Supplementary file 1 — Additional file 1: Figure S1. Diagram of the experimental set-up, Figure S2. Fermentation protocol, Figure S3. Total oxygen consumption during glucose metabolization, Figure S4. Determination of growth rate µ, Figure S5. Statistical significance of pairwise comparison of biomass yields YX/S, Calculation of theoretical RQ values. [file 13036_2024_453_MOESM1_ESM.pdf]

Supplementary figures:

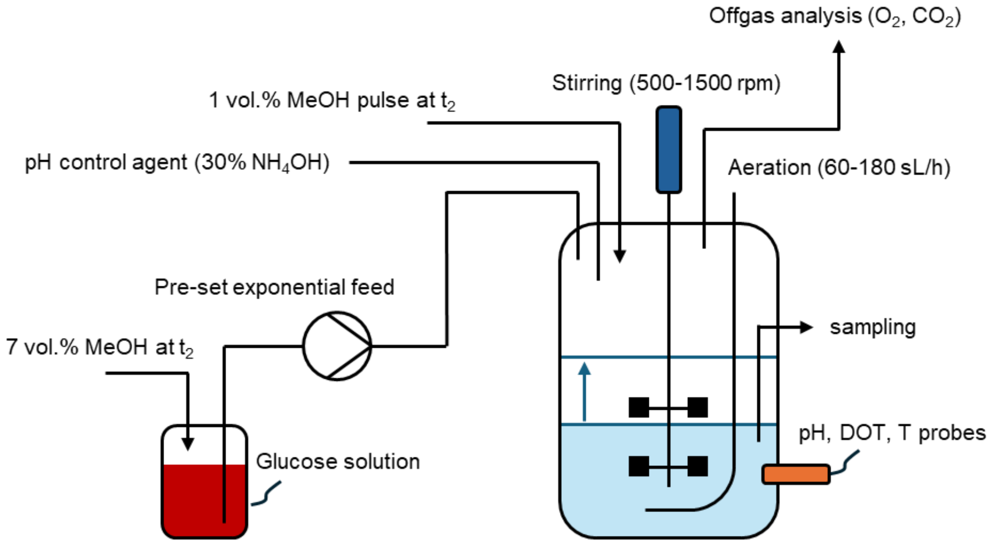

Figure S1: Diagram of the experimental set-up.

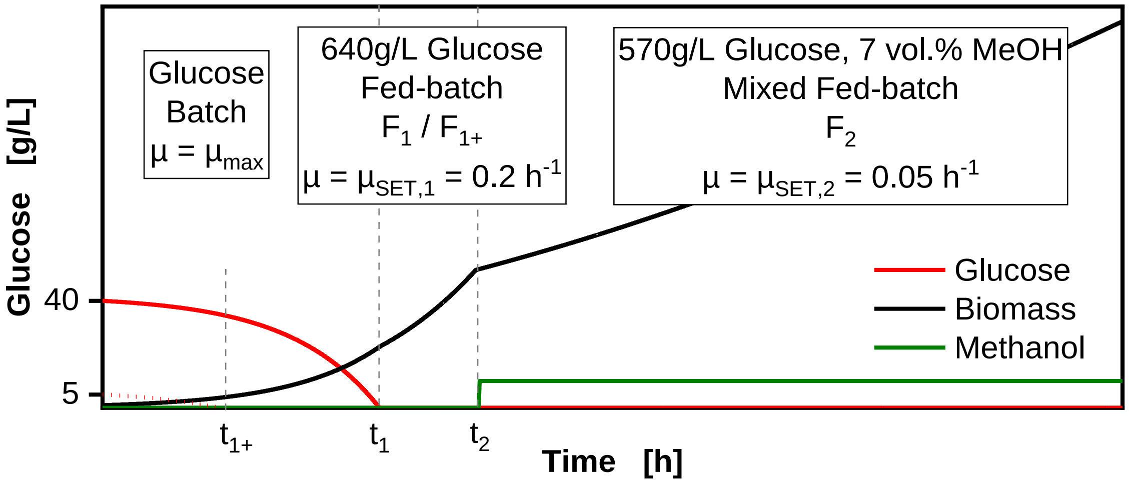

Figure S2: Fermentation protocol. Substrate and Biomass shown qualitatively over time.

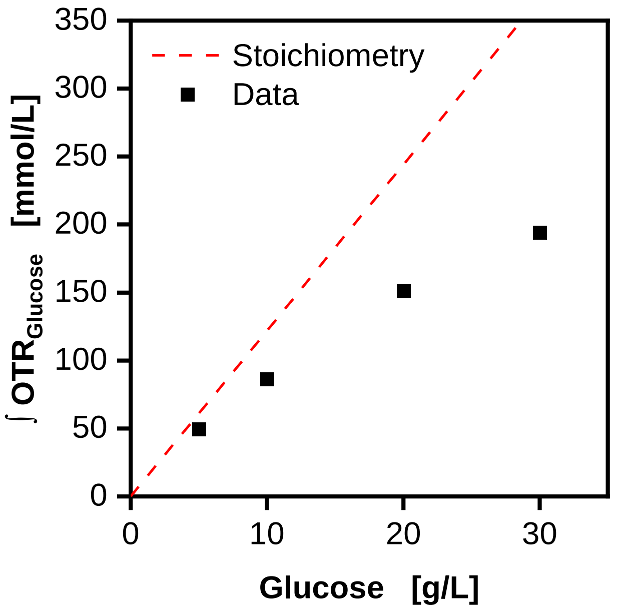

Figure S3: Total oxygen consumption during glucose metabolisation calculated from the integral of the first OTR peak in Figure 3. Stoichiometry calculated from equation 10. $Y_{O/S}$ = 12.2 mmol/g.

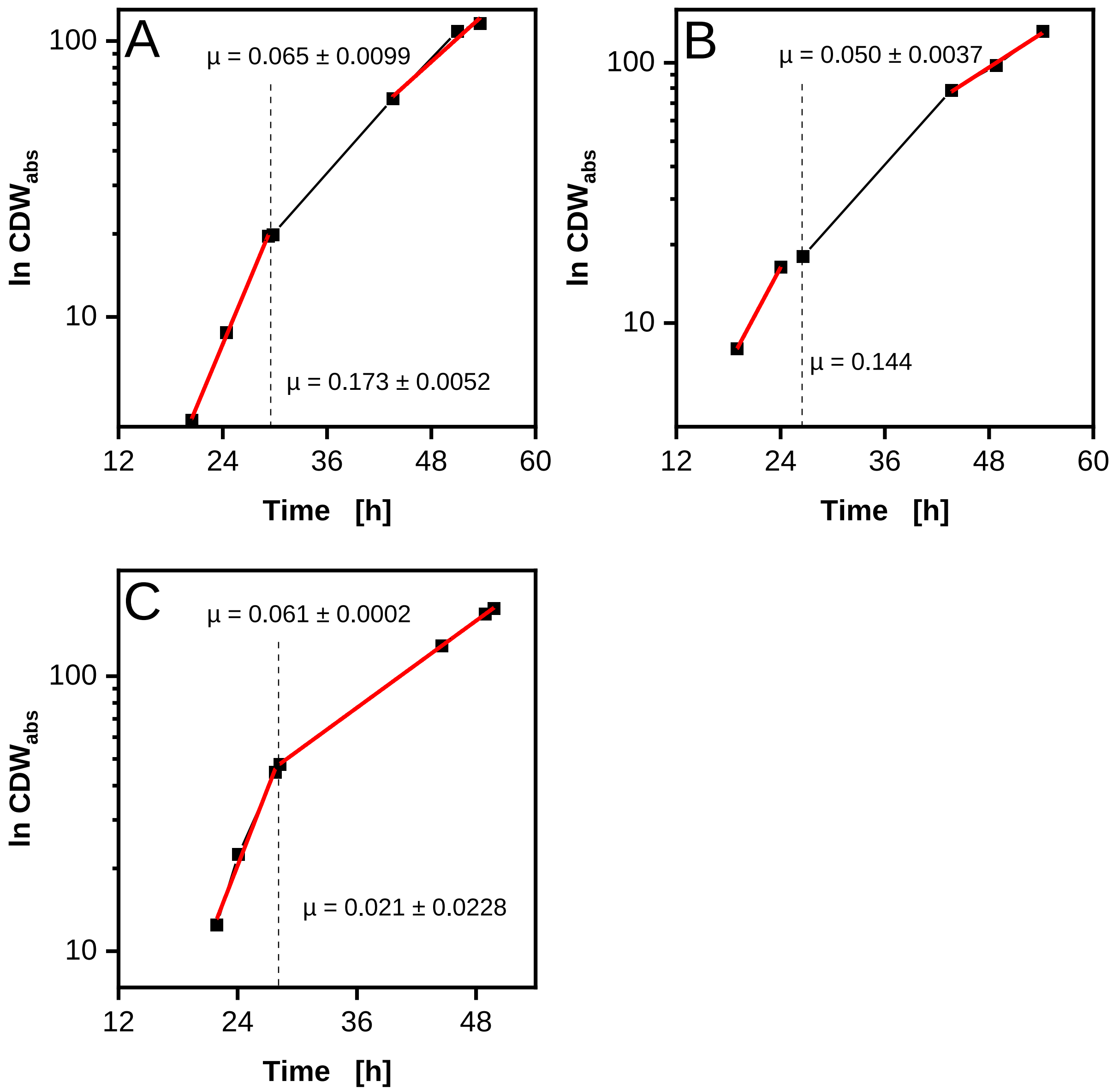

Figure S4: Determination of growth rate μ from absolute biomass CDW_{abs} data for BG11 strain. (A) shows CDW data for Glc. 40g/L DOT 30% fermentation depicted in Figure 1. (B) shows CDW data for Glc. 40g/L DOT 60% fermentation depicted in Figure 2. (C) shows data for Glc. 5g/L DOT 30% fermentation depicted in Figure 4. Data is interpolated linearly in each process phase. Induction is marked with a dashed line.

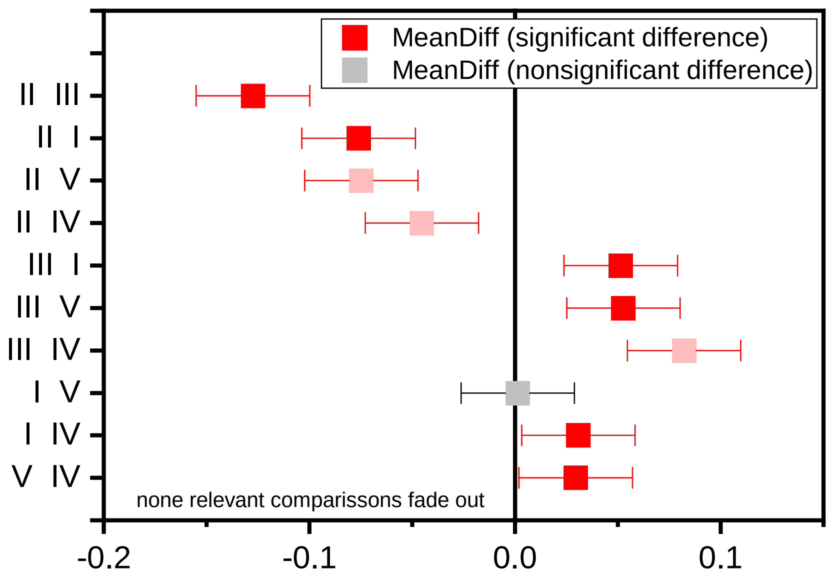

Figure S5: Statistical significance of pairwise comparison of biomass yields $Y_{X/S}$. Significance level of α=0.05. I) BG11 Glc. 40 g/L DOT 30%, II) BG11 Glc. 40 g/L DOT 60%, III) BG11 Glc. 5 g/L DOT 30%, IV) Prod. Glc. 40 g/L DOT 30%, V) Prod. Glc. 5 g/L DOT 30%.

Calculation of theoretical RQ values:

Theoretical RQ values were determined for full combustion of the metabolites.

Acetate: RQ = v(CO_2) / v(O_2) = 1.0

$$C_2H_4O_2 + 2\ O_2 \rightarrow 2\ CO_2 + 2\ H_2O \tag{1}$$

Ethanol: RQ = v(CO_2) / v(O_2) = 0.67

$$C_2H_6O + 3\ O_2 \rightarrow 2\ CO_2 + 3\ H_2O \tag{2}$$

Arabitol: RQ = v(CO_2) / v(O_2) = 0.91

$$C_5H_{12}O_5 + 5.5\ O_2 \rightarrow 5\ CO_2 + 6\ H_2O \tag{3}$$

Methanol: RQ = v(CO_2) / v(O_2) = 0.67

$$CH_4O + 1.5\ O_2 \rightarrow\ CO_2 + 2\ H_2O \tag{4}$$
